# Targeting lipid metabolism in cancer: neuroblastoma

**DOI:** 10.1007/s10555-022-10040-8

**Published:** 2022-06-10

**Authors:** Massimiliano Agostini, Gerry Melino, Bola Habeb, Jorgelina M. Calandria, Nicolas G. Bazan

**Affiliations:** 1 Department of Experimental Medicine, TOR, University of Rome Tor Vergata, 00133 Rome, Italy; 2 Neuroscience Center of Excellence, School of Medicine, Louisiana State University Health New Orleans, New Orleans, LA 70112 USA

**Keywords:** ELOVL4 transcription, Elovanoids, Very-long-chain polyunsaturated fatty acids, MYC, Fatty acid oxidation, Fatty acid synthesis

## Neuroblastoma

Neuroblastoma (NB) is the most common extracranial tumor among children with an average age of 17 months. NB is a tumor of the autonomic nervous system originating from embryonic neural crest cells [1] in which the pathogenesis of this malignancy is characterized by a block of differentiation [2, 3]. Many factors converge in this heterogeneous disease, including age, disease stage, and genetic and molecular features that, in turn, influence whether NB will spontaneously regress or metastasize and become resistant to therapy [4, 5]. Among the genetic alterations described in NB, MYCN amplification is the most common genetic dysfunction and is also associated with poor outcome. Moreover, mutations affecting both the α-thalassemia/mental retardation syndrome X-linked (*ATRX*) gene [6] or anaplastic lymphoma receptor tyrosine kinase (*ALK*) [7] are also common in NB.

Current therapeutic strategies for NB are selected according to patient stratification in four prognostic groups: low, intermediate, high risk, and tumor stage 4 [8]. Patients with low-risk disease (stage 1) and 4S0 are subjected to surgery alone. Patients with intermediate-risk (stages 2) first receive chemotherapy followed by resectioning of the tumor mass. Overall, in both prognostic categories, the survival is greater than 90%. In contrast, patients with high-risk disease (stages 3–4) are treated with dose-intensive chemotherapy plus radiotherapy. In addition, these patients undergo differentiation therapy with isotretinoin and immunotherapy with antidisialoganglioside GD2 monoclonal antibodies [9]. However, even with this aggressive treatment, children have the lowest overall survival (40–50%) and may also face a reduced quality of life. Therefore, it is urgent to develop novel therapeutics for less toxic and effective therapies. At the molecular level, several genes have been implicated in the pathogenesis of the disease, including the family of p53 [10–15], and in particular p73 [16–18], redox regulators [19–21], or apoptotic regulators [22]. However, MYCN amplification and activation of its downstream signaling is the most robust clinical biomarker of the poor clinical outcome and is present in about 40% of high-risk cases [23]. Consequently, targeting MYCN downstream cellular processes, including metabolism, may be exploited as a potent strategy to overcome the difficulties of directly targeting MYCN [24, 25].

## Metabolic alteration in neuroblastoma

Reprogramming of cellular metabolism is a hallmark of cancer. Indeed, cancer cells have deregulated glucose, lipids, and glutamine metabolism to sustain cell proliferation, control redox homeostasis, and overcome conditions of low nutrient and oxygen availability [26–30]. Positron emission tomography of cancer patients shows that NB tumors have high glucose uptake [31] and a high rate of lactic acid production, indicating the switch from OXPHOS to glycolysis [32]. In addition, a possible link between common genetic alterations in NB and metabolism has been explored. Deletion of the short arm of chromosome 1 (1p36) occurs in approximately 20–40% of primary NB [33]. Germline mutations of the succinate dehydrogenase enzymes complex (SDH), which is involved in the tricarboxylic acid cycle, are primarily predisposed to paraganglioma and phaeochromocytoma [34]. The gene encoding for the subunit B (SDHB) maps to the 1p36 region, and reduced mitochondrial activity has been described in undifferentiated neuroblastoma [35]. However, mutation analysis in 46 primary NB did not identify any germline or somatic SDHB mutations [36]. Gain of chromosome 17 is an additional genetic alteration in NB that correlates with high-stage disease and poor prognosis [37]. The antiapoptotic protein BIRC5/survivin, which maps to 17q25, is highly expressed in human NB and is associated with chemotherapy resistance and poor prognosis [38]. The underlying molecular mechanism associated with drug resistance is caused, in part, by a switch from oxidative phosphorylation to aerobic glycolysis [39].

Thus, MYCN amplification is another characteristic genetic alteration found in NB patients. MYCN protects NB cells from oxidative stress by increasing glutathione biosynthesis, and *in vivo* administration of glutathione biosynthesis inhibitors significantly potentiated the anticancer activity of cytotoxic chemotherapy against established tumors [40]. In addition, MYCN positively regulates the expression of solute carrier family 1 member 5 (ASCT2) to maintain sufficient levels of glutamine essential for the TCA cycle anaplerosis. Interestingly, glutamine transporter abundance correlates to poor prognosis in neuroblastoma patients [41]. In MYCN-amplified NB cells, MYCN cooperates with MondoA in regulating levels of proteins involved in lipid biosynthesis, and a subset of these proteins correlates with poor patient outcome [42]. Moreover, MYCN mediates structural changes in the mitochondrial network by increasing fusion, resulting in resistance to cell death induced by cisplatin [43].

Recently, the metabolic network regulated by MYCN has been further extended. Indeed, several observations have highlighted the involvement of MYCN in regulating lipid metabolism in NB cancer. Inhibition of MYCN and the downstream signaling pathway by several means in NB cells results in intracellular lipid droplet accumulation as a consequence of mitochondrial dysfunction [44]. Furthermore, lipid accumulation was shown to be caused mainly by inhibition of β-oxidation, suggesting that aggressive NB tumors use fatty acid as an energy source. This observation was further supported by analyzing the metabolic features of NB tumors with MYCN amplified. MYCN amplification enhances oxidative phosphorylation in NB cells [45], and more importantly, gene expression profile and proteomic analysis in patients show that high levels of MYCN are associated with elevated expression of key enzymes involved in glycolysis, Krebs cycle, and electron transport chain proteins. Patients with high expression of these genes show poor overall survival.

## Selective targeting lipid metabolism as therapeutic approach in neuroblastoma

Cancer cells are particularly dependent on lipid metabolism for energy production because they are key components of cellular membranes, storing precursors of biologically active lipid mediators [51–53]. Therefore, novel therapeutic strategies have been explored to inhibit lipid metabolism for improving clinical outcomes of cancer treatment [54]. In agreement with this, several clinical trials have assessed the possibility of inhibiting cholesterol biosynthesis by statins as a novel antitumor strategy [55]. However, this therapeutic approach has generated conflicting results [56, 57].

### Inhibition of fatty acid oxidation

Several tumors use fatty acids as a source of mitochondrial energy production [58]. In NB, MYCN amplification correlates with high expression of key genes involved in the regulation of fatty acid oxidation (FAO), including hydroxyacyl-CoA dehydrogenase (HADH), indicating that MYCN-amplified NB tumors are more dependent on OXPHOS compared to non-MYCN-amplified tumors [45]. Interestingly, high expression of some of these enzymes correlates with poor prognosis in NB patients, suggesting that fatty acids are a major substrate for OXPHOS-based energy metabolism in NB. Carnitine palmitoyl-transferase 1a (CPT1a) is the β-oxidation rate-limiting enzyme, and high expression of the gene correlates with poor prognosis in NB patients. Etomoxir is a small-molecule irreversible inhibitor of CPT1a used widely in preclinical studies. Recently, it has been shown that etomoxir treatment was able to reduce *in vivo* tumor growth of MYCN-amplified NB cells. Although, in phase II clinical trials, etomoxir has shown hepatic toxicity and has been suspended, these experiments are proof of concept that inhibition of FAO could be used for the development of novel therapeutic strategies. In addition, the novel reversible CPT1a inhibitor, teglicar, which was recently developed, does not show as severe toxicity as etomoxir [59]. Further studies are needed to assess the possibility of using teglicar for cancer treatment.

### *Inhibition of de novo fatty acid synthesis*

One common feature shared by almost all tumors is the reactivation of fatty acid synthesis (FAS) to support cancer cell proliferation, as cancer cells need lipids both as membrane components and as signaling molecules involved in cell homeostasis, cell death, and metastasis [54]. FAS, which takes place in cytoplasm, is regulated primarily by acetyl-CoA carboxylase (ACACA) and fatty acid synthase (FASN). Small-molecule inhibitors of these two enzymes, including TOFA and Soraphen A, target ACACA, and Cerulenin, Orlistat, and UB006 that target FASN are available and used largely in preclinical experiments that have shown promising antitumor activity [60]. Using these five inhibitors in different experimental approaches, including PDX-derived cell cultures and xenograft model of NB, it has been shown that inhibition of FAS resulted in decreased cell proliferation, reduced MYCN protein levels, and induction of neural differentiation [61]. Of note, these antitumor effects were independent from MYCN status. Among the inhibitors tested, only Orlistat has been approved by the FDA, although not for cancer treatment. Therefore, these observations strongly support the idea of developing more specific FAS inhibitors that can be used in the clinic.

### Differentiation therapy (elovanoids)

The highest-risk group of patients is often treated with isotretinoin (13-*cis*-retinoic acid) to induce terminal differentiation of NB cells, thus reducing the risk of relapse [62]. However, the benefits of using retinoids are uncertain [8, 63]. Therefore, it would be important to develop novel agents able to induce differentiation for treatments of NB. In this respect, elovanoids (ELVs) are a novel class of endogenous lipid mediators that protect against excitotoxicity and cell damage and modulate neuronal homeostasis [47, 50]. Recently, it has also been shown that ELV-N34:6 may have pharmacological activity against glioblastoma multiforme (GBM). Indeed, in the orthotopic model of GBM treatment with LAU-0901 (a platelet-activating factor receptor antagonist), ELV-N34:6, and Avastin (angiogenesis inhibitors) individually and with all three compounds in combination resulted in a reduction of tumor size [64, 65]. Therefore, since ELV-N34:6 has shown *in vivo* pharmacological activity, it is possible to hypothesize that ELV-N34:6 may be exploited for NB treatment in alternative or in combination with isotretinoin for inducing neural differentiation and improving patient outcome.

**Fig. 1:**
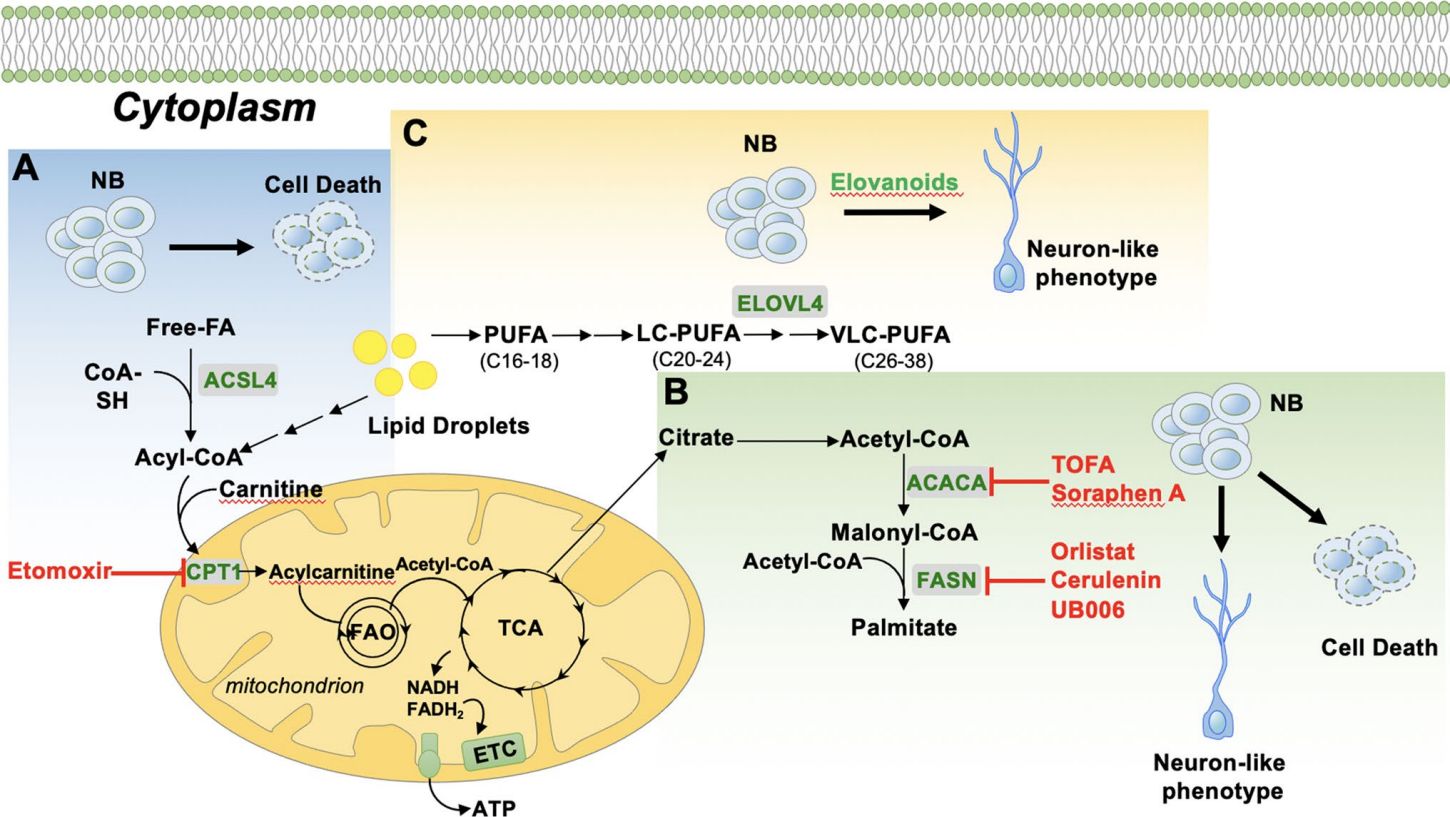
Novel potential pharmacological approaches in neuroblastoma treatment. **A** MYCN-amplified neuroblastoma (NB) cells rely on fatty acid–dependent mitochondrial respiration. Inhibition of fatty acid oxidation (FAO) with inhibitor targeting carnitine palmitoyl-transferase 1a (CPT1) reduces tumor growth by inducing cell death. **B** Reactivation of fatty acid synthesis (FASN) in cancer cells is required for providing lipid building blocks for sustained cell proliferation. Inhibition of FASN by small molecules decreases cell proliferation and results in neural differentiation of NB cells. **C** Elongation of very-long-chain fatty acids–4 (ELOVL4) is required for the proper neural differentiation of NB cells (Rugolo et al. 2021). We hypothesize that NB cells treated with elovanoids (ELVs) may induce differentiation of NB cells. NB: neuroblastoma; ACSL4: acyl-CoA synthetase long-chain family member 4; TCA: tricarboxylic acid cycle; ETC: electron transport chain; ACACA: acetyl-CoA carboxylase alpha; FASN: fatty acid synthase; PUFA: polyunsaturated fatty acid; LC-PUFA: long-chain polyunsaturated fatty acid; VLC-PUFA: long-chain poly unsaturated fatty acid

Very-long-chain polyunsaturated fatty acids (VLC-PUFA) play an important role in the maintenance of the homeostasis of several tissues, including neural tissue [46]. Among the family of PUFA products [47], ELVs represent a class of recently characterized lipid mediators that sustain cellular integrity against hemostasis disturbances [48, 49]. The enzyme ELOVL4 (elongation of VLC fatty acids–4), which is responsible for the elongation of > C28 FAs, mediates the biosynthesis of the precursors of elovanoids (ELV-N32 and ELV-N34). The expression of ELOVL4, which is repressed by MYCN, increases during neuronal differentiation of NB cells, and its presence is necessary for the progression of the differentiation. In addition, ELOVL4 expression is required to increase the lipid droplet number and VLC-PUFAs in differentiated cells [50]. Interestingly, more differentiated tumors (low- and intermediate-risk) show higher expression of ELOVL4 when compared with those less differentiated tumors (high-risk), and high levels of ELOVL4 identify subsets of NB patients with a better prognosis. Therefore, these observations suggest that dysregulation of ELOVL4 expression may participate in the lipid alterations that occur in NB. Overall, there is compelling experimental evidence that cellular metabolism, including lipid metabolism, is deregulated in NB cells and may therefore provide a novel, potential therapeutic target (see Fig. 1). Here we discuss some possible clinical applications of targeting lipid metabolism in NB that may be explored for therapeutic purposes.

## Data Availability

N/A.

## Code availability

N/A.
